# Changes in lipid abundance are associated with disease progression and treatment response in chronic *Trypanosoma cruzi* infection

**DOI:** 10.1186/s13071-024-06548-3

**Published:** 2024-11-09

**Authors:** Juan Carlos Gabaldón-Figueira, Albert Ros-Lucas, Nieves Martínez-Peinado, Gavin Blackburn, Irene Losada-Galvan, Elizabeth Posada, Cristina Ballart, Elisa Escabia, Jordi Capellades, Oscar Yanes, María-Jesús Pinazo, Joaquim Gascón, Julio Alonso-Padilla

**Affiliations:** 1 ISGlobal, Barcelona, Spain; 2 Facultat de Medicina i Ciències de la Salut, Universitat de Barcelona (UB), Barcelona, Spain; 3 CIBER de Enfermedades Infecciosas, Instituto de Salud Carlos III (CIBERINFEC, ISCIII), Madrid, Spain; 4 Universitat Pompeu Fabra (UPF), Barcelona, Spain; 5 Secció de Parasitologia, Departament de Biologia, Sanitat i Medi Ambient, Facultat de Farmàcia i Ciències de l’Alimentació, Universitat de Barcelona, 08028 Barcelona, Spain; 6 MVLS Shared Research Facilities, University of Glasgow, Glasgow, G12 8QQ Scotland; 7 Institut d’Investigació Sanitària Pere Virgili (IISPV), Metabolomics Platform, Reus, Spain; 8 Department of Electronic Engineering, Universitat Rovira i Virgili, Tarragona, Spain; 9 CIBER de Diabetes y Enfermedades Metabólicas Asociadas (CIBERDEM), Instituto de Salud Carlos III, Madrid, Spain; 10 Drugs for Neglected Diseases Initiative (DNDi), Rio de Janeiro, Brazil

**Keywords:** *Trypanosoma cruzi*, Chagas disease, Treatment response, Metabolomics, Lipidomics, Phosphatidylethanolamine, Hydroxydecanoic acid, Sphingolipids

## Abstract

**Background:**

Chagas disease, caused by the parasite *Trypanosoma cruzi*, is a zoonosis that affects more than seven million people. Current limitations on the diagnosis of the disease hinder the prognosis of patients and the evaluation of treatment efficacy, slowing the development of new therapeutic options. The infection is known to disrupt several host metabolic pathways, providing an opportunity for the identification of biomarkers.

**Methods:**

The metabolomic and lipidomic profiles of a cohort of symptomatic and asymptomatic patients with *T. cruzi* infection and a group of uninfected controls were analysed using liquid chromatography/mass spectrometry. Differences among all groups and changes before and after receiving anti-parasitic treatment across those with *T. cruzi* infection were explored.

**Results:**

Three lipids were found to differentiate between symptomatic and asymptomatic participants: 10-hydroxydecanoic acid and phosphatidylethanolamines PE(18:0/20:4) and PE(18:1/20:4). Additionally, sphinganine, 4-hydroxysphinganine, hexadecasphinganine, and other sphingolipids showed post-treatment abundance similar to that in non-infected controls.

**Conclusions:**

These molecules hold promise as potentially useful biomarkers for monitoring disease progression and treatment response in patients with chronic *T. cruzi* infection.

**Graphical Abstract:**

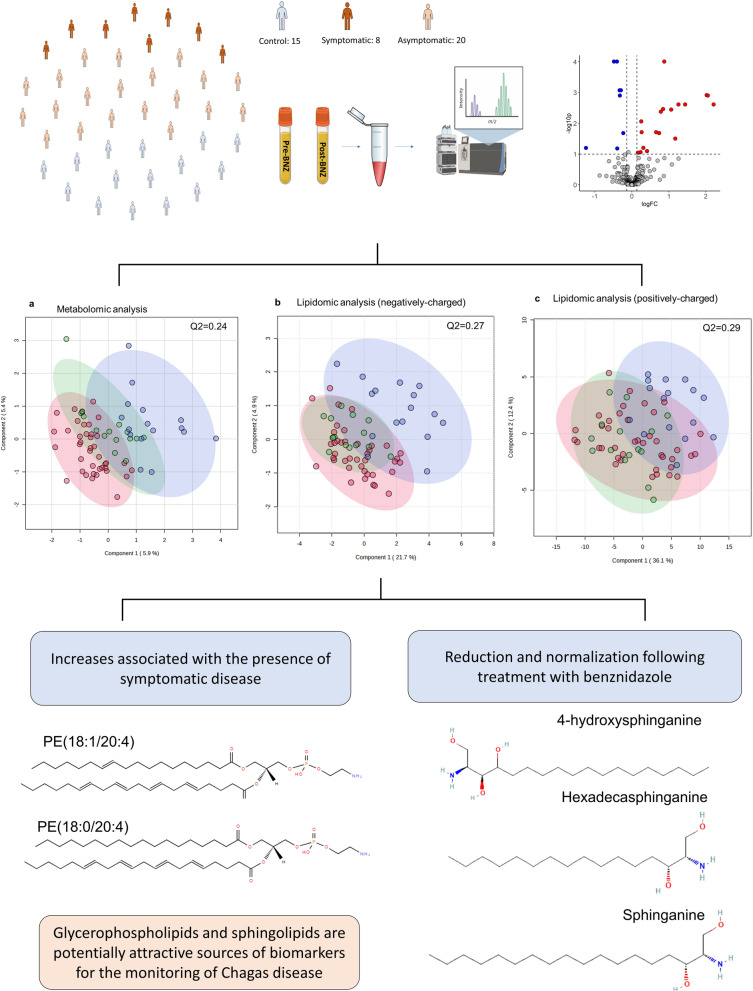

**Supplementary Information:**

The online version contains supplementary material available at 10.1186/s13071-024-06548-3.

## Background

Chagas disease (CD), caused by the protozoon parasite *Trypanosoma cruzi*, is a largely neglected zoonosis estimated to affect seven million people and to be responsible for approximately 9000 deaths every year [1]. Symptomatic disease will appear in 30–40% of those chronically infected and can lead to potentially lethal cardiac and digestive manifestations [1]. Changes in the expression of microRNAs [2] and a variety of other host-derived molecules [3, 4] have been identified as potential markers to predict the progression of organ damage.

More modest progress has been achieved in the identification of biomarkers of treatment response, which largely relies on the longitudinal serologic monitoring of patients for long periods of time [5]. At present, the cure criterion for chronic *T. cruzi* infection is seronegativization [6], a process that can take up to 30 years, depending on the stage of the disease [7]. This complicates the monitoring of patients and the design of clinical trials evaluating new therapeutic options [8], making the identification of new markers of response to treatment an unmet medical need [8].

*Trypanosoma cruzi* infection disrupts several metabolic pathways, including fatty acid (FA) oxidation, phospholipid synthesis, glycolysis, and the catabolism of amino acids [9], which are attractive targets for the identification of novel biomarkers.

Perturbations in steroidogenesis and peptide metabolism have also been linked to the pathogenesis of chronic disease [10, 11], and a recent metabolomic study of tissue samples from patients with end-stage heart failure revealed changes in amino acid, FA, and glycerophospholipid metabolism [12]. We used similar methods to further characterize metabolic changes associated with the development of chronic symptomatology as well as the response to anti-parasitic treatment in a cohort of participants with chronic *T. cruzi* infection.

## Methods

### Clinical data and sample collection

Study participants were enrolled at the Hospital Clinic of Barcelona (HCB; Spain) between May 2019 and June 2020. Participants were tested for *T. cruzi* infection using two serologic assays: enzyme-linked immunosorbent assay (ELISA) (Vircell Chagas ELISA IgG+IgM, Granada, Spain) and CMIA ARCHITECT Chagas (Abbot, Wiesbaden, Germany). Infected participants were also tested using an in-house real-time polymerase chain reaction (rtPCR) assay [13], before and after completing anti-parasitic treatment. They underwent further clinical examination as well, including electrocardiography, transthoracic echocardiography, and/or chest X-ray. Additional radiological evaluation was performed on patients with digestive symptomatology. Infected participants were classified as symptomatic if any Chagas-specific cardiac or digestive alterations were detected.

### Sample processing

Venous blood was collected in ethylenediaminetetraacetic acid (EDTA) tubes. For those with infection, samples were obtained before treatment and, on average, 8 months after completing standard anti-parasitic treatment with benznidazole (5 mg/kg/day for 60 days). Plasma was segregated within the first hour of collection and stored at −80 ºC until further processing. For the untargeted metabolomic analysis, plasma samples were thawed for 1.5 h and extracted using a chloroform/methanol/water mixture (1:3:1 ratio). Following centrifugation at +4 ºC, 200 µl of the samples was stored at −80 ºC until processing for liquid chromatography/mass spectrometry (LC/MS). A similar approach was used for the lipidomic analysis, but using isopropanol as extraction solvent and centrifuging for 10 min at +4 ºC. Pools were made for the quality control of both analyses.

### LC/MS setup, metabolite quantification, and identification

The LC/MS analysis of the metabolomic samples was performed on a Dionex UltiMate 3000RSLC system connected to an Orbitrap Fusion mass spectrometer (Thermo Fisher Scientific, Hemel Hempstead, UK). Separation was achieved with a ZIC-pHILIC column (150 mm × 4.6 mm, 5 µm column, Merck SeQuant). Mobile phase A was 20 mM ammonium carbonate and mobile phase B was acetonitrile. The LC/MS run for each sample was conducted with the following gradient: 0–15 min, 80–20% B; 15–17 min, 5% B; 17–26 min, 80% B. The MS was run in positive/negative switching mode to acquire both positive and negative ions for each sample within the same run. Data were acquired at a resolution of 120,000 and across a mass range of 70–1000 atomic mass units (amu). Raw files from the instrument were converted to mzXML files via MSConvert [14] and uploaded to Polyomics integrated Metabolomics Pipeline (PiMP) [15]. Once uploaded, the data were processed using the standard pipeline. Briefly, peaks were picked using eXtensible Computational Mass Spectrometry (XCMS) combined by sample group [16], filtered for noise and on relative standard deviation (SD), recombined as a total set, gap-filled, and passed on for further processing. For the lipidomic analysis, LC/MS was performed with a Vanquish Horizon ultra-high-performance liquid chromatography (UHPLC) system (Thermo Scientific, Waltham, MA, USA) interfaced with an Orbitrap ID-X Tribrid mass spectrometer (Thermo Scientific, Waltham, MA, USA). Lipids were separated by reversed-phase chromatography with an Acquity UHPLC C18-RP (ACQUITY UPLC BEH C18 1.7 µM, Waters). Mobile phase A was acetonitrile/water (60:40) (10 mM ammonium formate), and mobile phase B was isopropanol/acetonitrile (90:10) (10 mM ammonium formate). Separation was conducted under the following gradient: 0–2 min, 15–30% B; 2–2.5 min, 48% B; 2.5–11 min, 82% B; 11–11.5 min, 99% B; 11.5–12 min, 99% B; 12–12.1 min, 15% B; 12.1–15 min, 15% B. For MS detection, heated electrospray ionization settings were set in positive and negative ionization modes and a resolution of 120,000. Data were acquired across a mass range of 180–1800 amu. The mzXML files were further processed and annotated using RHermes software [17].

### Statistical analysis

All statistical analysis was performed in Metaboanalyst 5.0 [18] or RStudio version 2023.06.1+524. Processed data were log10-adjusted and analysed using Metaboanalyst 5.0 [18]. A partial least-squares discriminant analysis (PLS-DA) was used for an initial evaluation of trends in the data, and to identify unexpected variability. The accuracy of this model was determined by estimating its *Q*^2^ value [19]. A *Q*^2^ value closer to 1 indicates a better predictive capacity. Differences between clinical groups were initially compared using a one-way analysis of variance (ANOVA). Given the demographic differences between the study groups, we used the limma R package [20] to run a multiple linear regression adjusted for sex and age to estimate fold changes (FC) and *P*-values for each feature and comparison. Additionally, when pre- and post-treatment samples were included in the comparisons, the identification (ID) of each participant was blocked and treated as a random effect to account for the inclusion of repeated samples from a single participant in the model. A paired *t*-test was used to compare pre- and post-treatment groups. All *P*-values were adjusted for multiple comparisons using the Benjamini–Hochberg method to control the false discovery rate (FDR). Given the exploratory nature of the study, differentially abundant features were defined as those with a log2 fold change (logFC) ≥ ±0.138 (corresponding to an FC of 1.1) and an FDR below 10% (adjusted *P*-value < 0.1). The geom_point, geom_boxplot, and geom_jitter functions of the ggplot2 package [21] were used to construct volcano and box-and-whisker plots. In the latter, the line represents the median and the box indicates the interquartile range. Receiver operating characteristic (ROC) curves were constructed in Metaboanalyst 5.0, which was also used to construct a PLS-DA algorithm for the classification model with a two-latent-variable input. The predictive accuracy of the model was estimated using the cross validation (CV) method and 1000 permutations [22].

## Results

### Characteristics of the cohort

Forty-three participants were recruited during the study period (Table 1). Samples were obtained from all infected participants (*n* = 28, 20 classified as asymptomatic and eight as symptomatic) before starting standard anti-parasitic treatment with benznidazole and, on average, 8 months (SD ±3.2 months) after the last dose. Fifteen non-infected participants from a similar geographical origin were included as controls. In total, 71 samples were processed for the metabolomic and lipidomic analyses (40 from asymptomatic participants, 16 from symptomatic participants, and 15 from controls). A full description of the clinical characteristics of this cohort has been provided elsewhere [23]. While several participants had a history of comorbidities (Table 1), these had been successfully treated and the patients were asymptomatic at the time of inclusion in the study.

**Table 1:** Characteristics of the cohort

	Asymptomatic	Symptomatic	Control	*P*-value
Demographics				
Number of participants (samples)	20 (40)	8 (16)	15 (15)	–
Sex (male/female)	5/15	1/7	6/9	0.39
Weight in kg (mean ± SD)	68.2 ± 10.4	70.4 ± 8.8	65.3 ± 12.2	0.59
Age (median, [range])	46.5 [23–60]	55.2 [40–64]	38.7 [25–53]	< 0.001
rtPCR (positive/tested)	7/20	2/8	0/15	0.02
Geographical origin				
Bolivia	19	7	11	–
Honduras	1	0	3	–
Brazil	0	1	0	–
Paraguay	0	0	1	–
Comorbidities				
Strongyloidiasis	2	0	1	–
Neurocysticercosis	1	0	0	–
*Helicobacter pylori* infection	1	0	0	–
Chronic arthrosis	1	0	0	–
Hypothyroidism	0	1	0	–
Lymphoproliferative syndromes	0	2	0	–
Asthma	0	1	0	–

rtPCR: real-time polymerase chain reaction; SD: standard deviation

Continuous data are presented as mean/SD or median/range and compared using either a one-way analysis of variance (ANOVA) or a Kruskal–Wallis test. Categorical data are presented as counts and compared using Fisher’s exact test

### Metabolic profiles differ between clinical groups

**Fig. 1:**
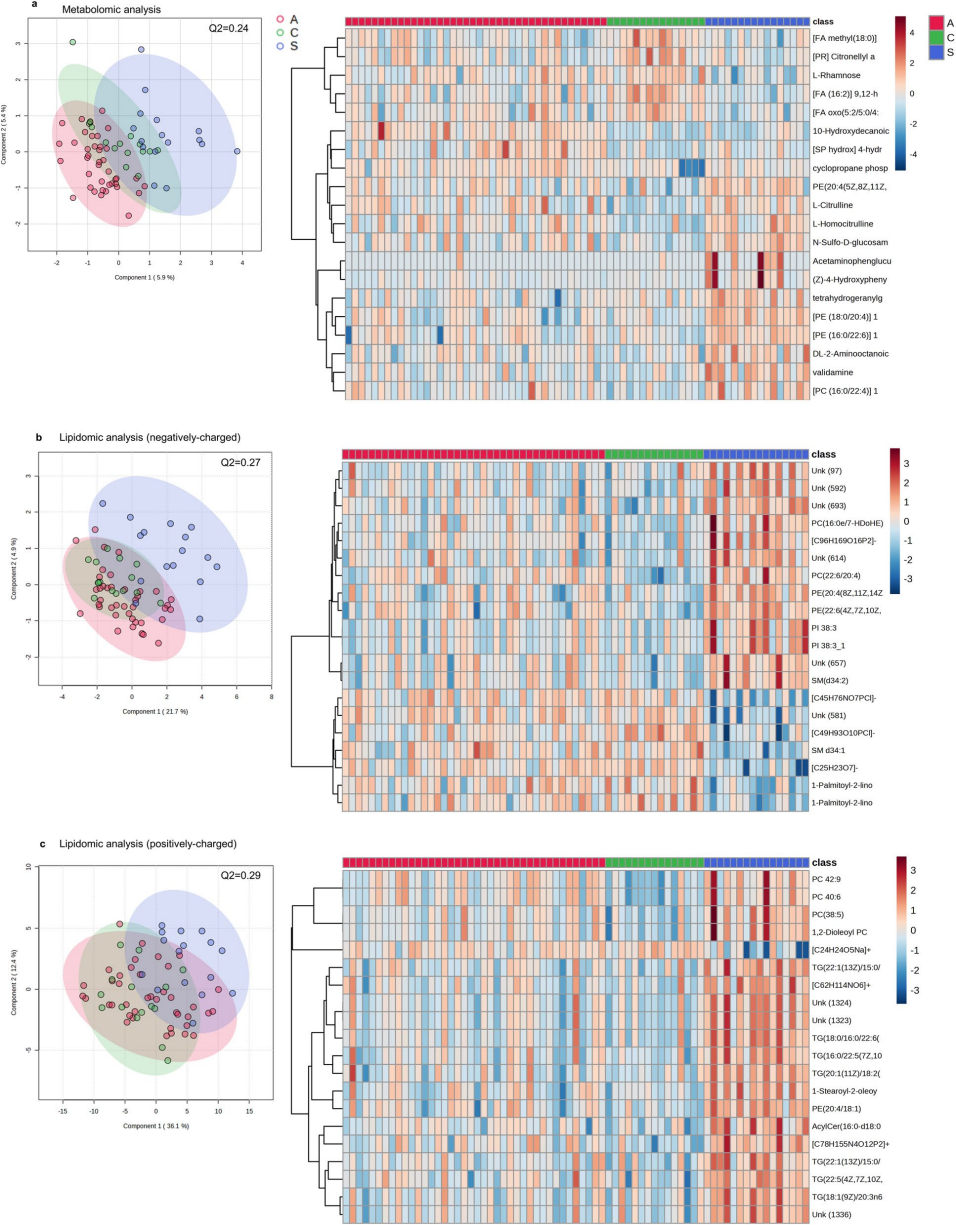
General metabolic trends observed in the clinical groups. PLS-DA analysis showing the separation between controls (*n* = 15 samples from 15 participants), asymptomatic (*n* = 40 samples from 20 participants), and symptomatic (*n* = 16 samples from eight participants) groups and heatmap of the top 20 most differentially abundant molecules (covariate-unadjusted one-way ANOVA) in the metabolomic (**a**), negatively charged, (**b**) and positively charged lipidomic analysis (**c**). Colours in the heatmap represent log10 peak intensity scale. In all cases: A stands for asymptomatic group, C for control group, and S for symptomatic group

In total, 442 features were annotated in plasma samples during the untargeted metabolomic analysis, which included pre- and post-treatment samples. PLS-DA showed a modest capacity to discriminate groups (*Q*^2^ = 0.24, with five components; Fig. 1a). While symptomatic and asymptomatic participants were adequately separated, significant overlap of both groups with controls was observed.

Changes were particularly evident in glycerophospholipids, including several phosphatidylethanolamines (PE) and phosphatidylcholines (PC), which were generally increased in the symptomatic group. On the other hand, free FAs were reduced in infected participants compared to controls, while 10-hydroxydecanoic acid was more abundant in asymptomatic participants than in the other two groups (Fig. 1a). Non-proteinogenic amino acids and their derivatives, including l-citrulline, l-homocitrulline, and validamine, were also increased in most symptomatic and some asymptomatic subjects, compared with controls (Fig. 1a).

**Fig. 2:**
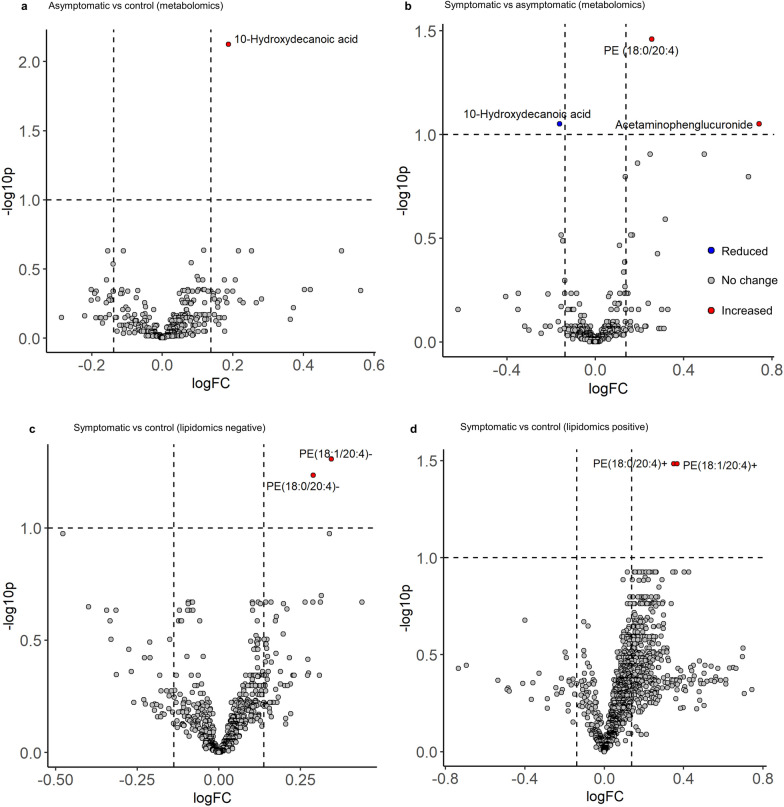
Differentially abundant metabolites in the clinical groups. Comparison of the metabolomic analysis of asymptomatic versus control participants (**a**). Comparison of symptomatic versus asymptomatic participants (**b**). Comparison of symptomatic participants versus controls in the negatively charged lipidomic analysis (**c**). Comparison of symptomatic group versus controls in the positively charged lipidomic analysis (**d**). Groups were compared using a multiple linear regression adjusted for sex and age, and treating participant ID as a random effect, to account for the inclusion of pre- and post-treatment samples. All *P*-values were adjusted using the Benjamini–Hochberg method to control the FDR. Features were considered to be differentially abundant if the logFC was >  ± 0.138, and had an FDR < 0.1

Upon covariate adjustment, only 10-hydroxydecanoic acid was more abundant in the asymptomatic group than in controls (Fig. 2a). No significant differences were observed between controls and the symptomatic group. In contrast, two metabolic features, PE(18:0/20:4) and acetaminophen glucuronide, were significantly more abundant in symptomatic than in asymptomatic participants, while 10-hydroxydecanoic acid was reduced in the former (Fig. 2b). Acetaminophen glucuronide is a by-product of acetaminophen metabolism, and is unlikely to be associated with CD. Complete results of the covariate-adjusted metabolomic model are presented in Additional file 1.

We observed increases in 10-hydroxydecanoic acid, sphinganine and 4-hydroxysphinganine (4-HS) in untreated asymptomatic participants compared with controls. The untreated symptomatic group had decreased D-glucarate compared with the asymptomatic group. PE(18:0/20:4) was increased in treated symptomatic participants when compared with the asymptomatic and control groups (Additional file 2). Complete comparisons between clinical groups disaggregated based on their treatment status, and controls are fully described in Additional files 2 and 3.

### Lipidomic analysis

Negatively and positively charged ions were reported independently, identifying 784 and 1495 different features, respectively. A moderate separation was observed between symptomatic and asymptomatic participants, with a large overlap of the latter and controls (Fig. 1b, c). The overall performance of these models was modest (*Q*^2^ = 0.27 and 0.29 for negatively and positively charged lipids, respectively).

*Negatively charged lipids*: Changes in glycerophospholipid abundance were confirmed in the lipidomic analysis, with an apparent enrichment of PE, PC, and phosphatidylinositol in the symptomatic group (Fig. 1b). Upon covariate adjustment, changes were only observed between symptomatic participants and controls, with a deprotonated form of the previously identified PE(18:0/20:4) and a deprotonated form of related PE(18:1/20:4), both being significantly more abundant in symptomatic participants (Table 2, Fig. 2c).

**Table 2:** Metabolic features differentially detected in participants with symptomatic *T. cruzi* infection

MM (g/mol)	RT (s)	Putative annotation	Formula	logFC	Adj. *P*-value
Metabolomic analysis					
Symptomatic versus asymptomatic					
767.55	176.69	PE (18:0/20:4)	C_43_H_78_NO_8_P	↑ 0.26	0.035
188.14	203.82	10-Hydroxydecanoic acid	C_10_H_20_O_3_	↓ −0.16	0.089
Lipidomic analysis					
Negatively charged					
Symptomatic versus control					
764.52	435.79	PE (18:1/20:4)	[C_43_H_75_NO_8_P]−	↑ 0.34	0.049
766.54	476.68	PE (18:0/20:4)	[C_43_H_77_NO_8_P]−	↑ 0.29	0.058
Positively charged					
Symptomatic versus control					
768.55	475.99	PE (18:0/20:4)	[C_43_H_79_NO_8_P]+	↑ 0.35	0.033
766.54	435.11	PE (18:1/20:4)	[C_43_H_77_NO_8_P]+	↑ 0.36	0.033

LogFC represents the log2 fold change in the mean abundance of a feature in the symptomatic versus the comparison group. *P*-values were obtained using multiple linear regression adjusted for age and sex, and treating participant ID as a random effect, to account for the inclusion of pre- and post-treatment samples; *P*-values account for multiple testing using the Benjamini–Hochberg method to control the FDR

MM: monoisotopic mass; RT: retention time; PE: phosphatidylethanolamine

*Positively charged lipids*: An apparent enrichment of triglycerides and certain PCs was observed in the symptomatic group (Fig. 1c). The protonated forms of PE(18:0/20:4) and PE(18:1/20:4) were again more abundant in symptomatic participants than in controls (Table 2, Fig. 2d). Full results of the covariate-adjusted model used in the lipidomic analysis are presented in Additional file 4.

### Changes in PE and 10-hydroxydecanoic acid abundance differentiate symptomatic participants

**Fig. 3:**
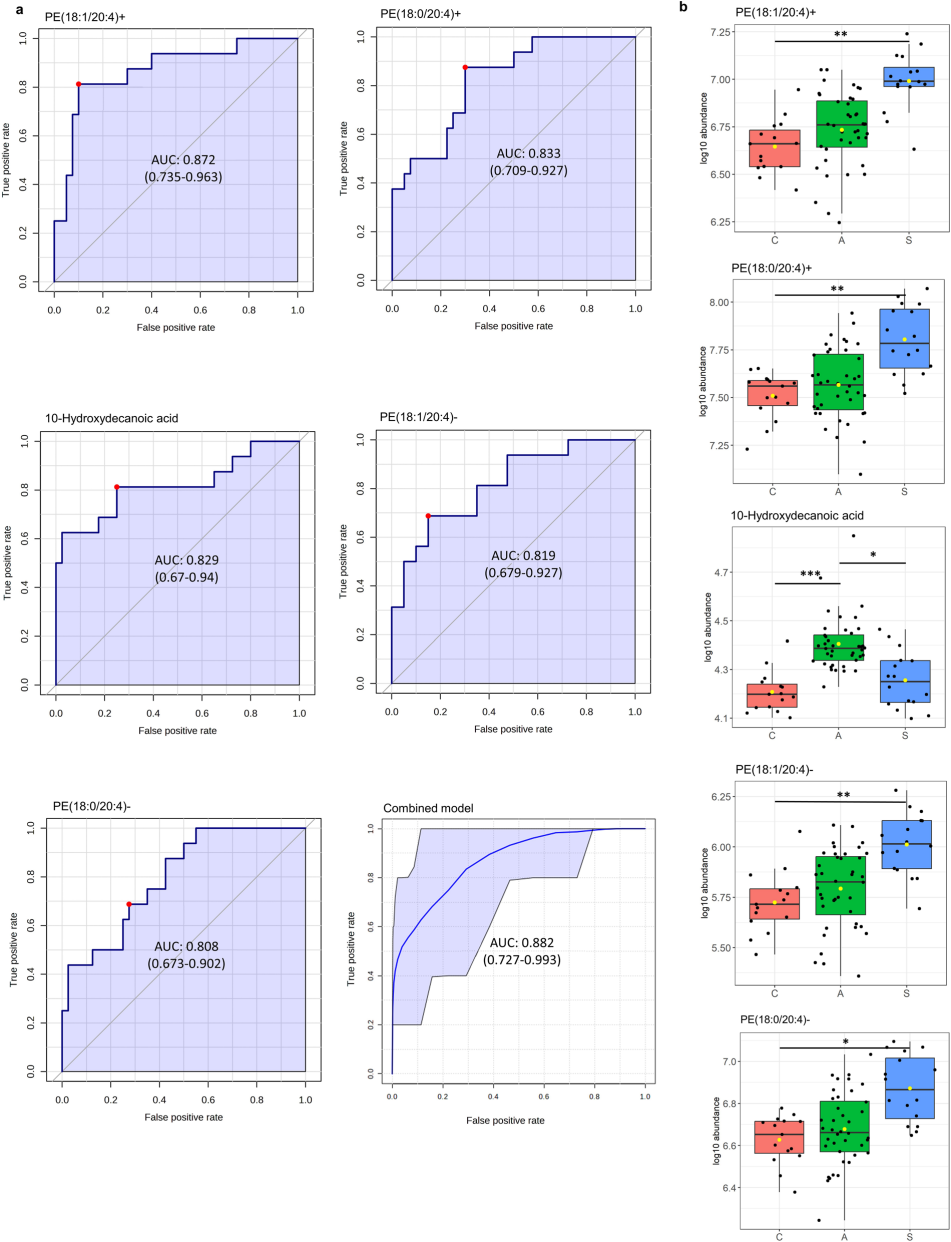
ROC curve analysis of features differentially abundant in symptomatic participants. ROC curves of the five differentially abundant molecules in symptomatic participants, and the combined model (**a**). Box-and-whisker plots of the abundance of metabolites in control (15 samples from 15 participants), asymptomatic (40 samples from 20 participants), and symptomatic participants (16 samples from eight participants) (**b**). In the box-and-whisker plots, lines represent the median, yellow dots represent group means, and boxes represent the interquartile range (IQR). Comparisons between different clinical groups were obtained using a multiple linear regression adjusted for sex and age and treating participant ID as a random effect, to account for the inclusion of pre- and post-treatment samples. All *P*-values were adjusted using the Benjamini–Hochberg method to control the FDR. In the statistical comparisons: *indicates 0.05 < *P* < 0.1, **indicates 0.01 < *P* < 0.05, and *** indicates *P* < 0.01. In all cases, A stands for the asymptomatic group, C for the control group, and S for the symptomatic group

In total, six features corresponding to three metabolites were found to be more abundant in symptomatic participants: 10-hydroxydecanoic acid, detected in the initial metabolomic analysis; PE(18:0/20:4), detected in the metabolomic and the two lipidomic analyses; and PE(18:1/20:4), which was detected in the two lipidomic analyses (Table 2, Fig. 3).

In order to evaluate the potential of these metabolites as biomarkers of symptomatic disease, we constructed individual ROC curves discriminating between symptomatic and asymptomatic participants. We included the positively and negatively charged forms of both PEs and 10-hydroxydecanoic acid. The positively charged forms of PE(18:1/20:4) and PE(18:0/20:4) performed best (area under the ROC curve [AUC] = 0.87 [95% CI 0.73–0.96] and AUC = 0.83 [95% CI 0.70–0.92], respectively). They were followed by 10-hydroxydecanoic acid (AUC = 0.83 [95% CI 0.67–0.94] and the negatively charged forms of both PEs (AUC = 0.82 [95% CI 0.68–0.92] and AUC = 0.80 [95% CI 0.67–0.90], respectively) (Fig. 3a).

We further constructed a PLS-DA classification algorithm with these features, which reached an AUC of 0.88 (95% CI 0.73–0.99) (Fig. 3a), and a predicted accuracy of 80% (*P* = 0.003 upon 1000 permutations; Additional file 5).

### Anti-parasitic treatment is associated with reductions in sphingolipid abundance among asymptomatic participants

**Fig. 4:**
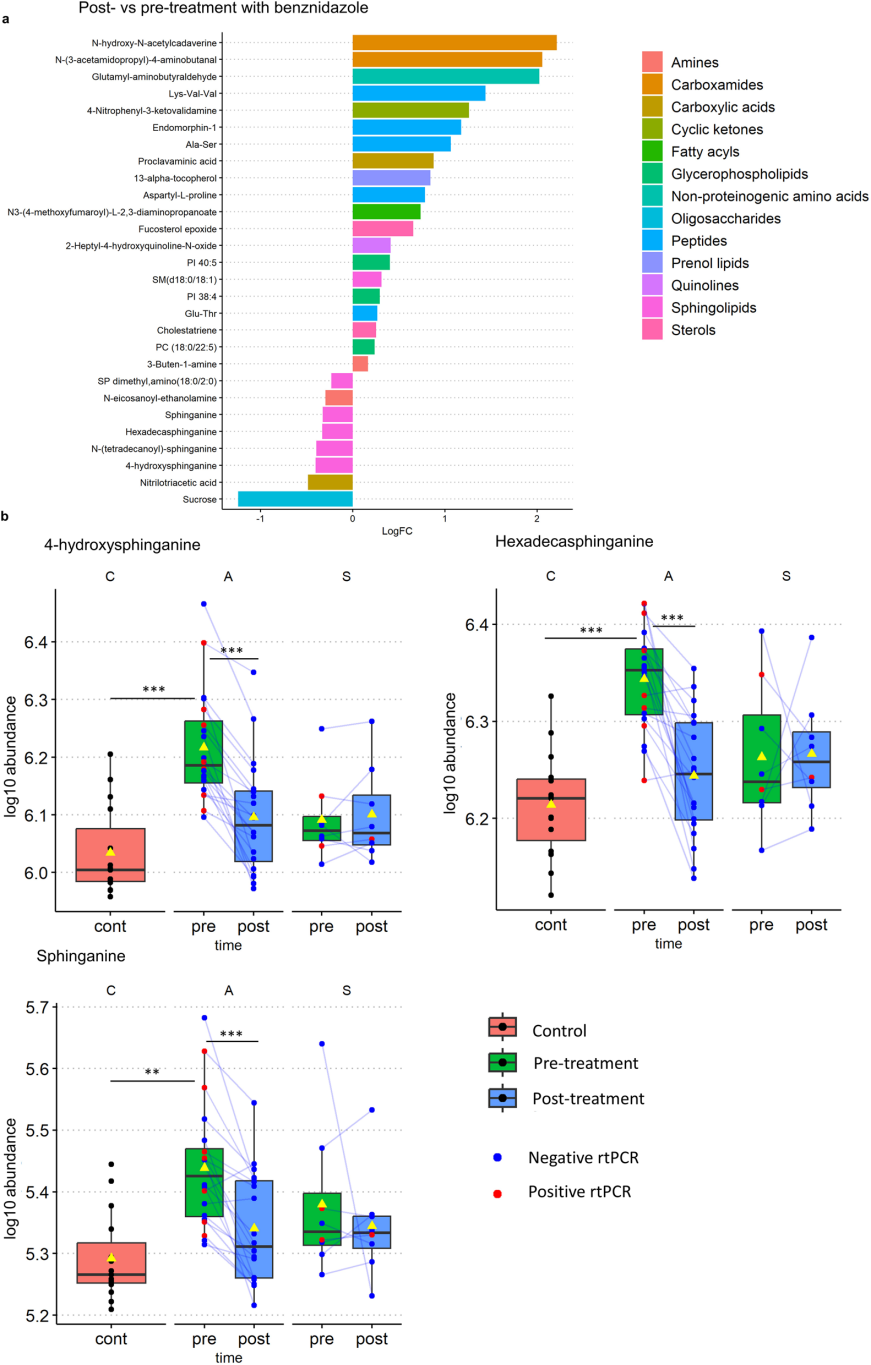
Metabolic changes observed following anti-parasitic treatment with benznidazole. Metabolites differentially abundant in the asymptomatic post-treatment group (**a**). Box-and-whisker plots of 4-hydroxysphinganine, hexadecasphinganine, and sphinganine in control (C, 15 samples), asymptomatic (A, 20 samples pre- and post-treatment), and symptomatic groups (S, 8 samples pre- and post-treatment) (**b**). Yellow triangles represent group means. *P*-values for comparisons between different clinical groups were obtained using a multiple linear regression adjusted for sex and age. Comparisons between pre- and post-treatment time points were obtained using a paired *t*-test. All *P*-values were adjusted using the Benjamini–Hochberg method to control the FDR: *indicates 0.05 < *P* < 0.1, **indicates 0.01 < *P* < 0.05, and ***indicates *P* < 0.01

We observed significant (logFC ≥  ± 0.138, *P* < 0.1) increases in 20 metabolites and reductions in eight features in the asymptomatic group. Differentially abundant metabolites included a variety of peptides and amino acid derivatives, sterols, prenol lipids, carboxylic acids, oligosaccharides, and sphingolipids (Fig. 4a**, **Additional file 6). The last group included five of the eight metabolites with reduced abundance after treatment: 4-HS, hexadecasphinganine, sphinganine, SP dimethyl, amino (18:0/2:0) 2S-(dimethylamino)-1,3R-octadecanediol, and [SP (14:0)] *N*-(tetradecanoyl)-sphinganine (Table 3).

**Table 3:** Sphingolipids differentially detected in samples from asymptomatic participants following anti-parasitic treatment

MM (g/mol)	RT (s)	Putative annotation	Formula	LogFC	Adj. *P*-value
317.29	249.78	4-Hydroxysphinganine	C_18_H_39_NO_3_	↓ −0.40	< 0.001
273.27	268.39	Hexadecasphinganine	C_16_H_35_NO_2_	↓ −0.33	0.001
301.30	258.21	Sphinganine	C_18_H_39_NO_2_	↓ −0.33	0.001
329.33	240.48	SP dimethyl, amino (18:0/2:0)	C_20_H_43_NO_2_	↓ −0.23	0.021
730.60	193.70	SM (d18:0/18:1)	C_41_H_83_N_2_O_6_P	↑ 0.31	0.063
511.50	179.30	[SP (14:0)] *N*-(tetradecanoyl)-sphinganine	C_32_H_65_NO_3_	↓ −0.39	0.066

LogFC represents the log2 fold change in the mean abundance of a feature in the post- versus the pre-treatment group.* P*-values were obtained using a paired *t*-test, and adjusted to account for multiple testing using the Benjamini–Hochberg method to control the FDR

MM: monoisotopic mass; RT: retention time; SM: sphingomyelin; SP: sphingolipid.

Post-treatment reductions in 4-HS, sphinganine, and hexadecasphinganine are remarkable, since these three molecules were significantly increased in untreated asymptomatic participants compared with controls (*t* = 5.38, *P* < 0.001 for 4-HS, *t* = 5.21, *P* < 0.001 for hexadecasphinganine, and *t* = 3.95, *P* = 0.03 for sphinganine), but not when the groups were compared after treatment (*t* < 1.6, *P* > 0.5 for the three metabolites) (Fig. 4b; Additional files 2 and 3). Contrary to other metabolites that also changed with treatment (Additional file 6), reductions seen in sphingolipids led to post-treatment abundance similar to that in non-infected controls (Fig. 4b). These changes were not observed in the symptomatic group, where only increases in proclavaminic acid were detected (Additional file 6).

## Discussion

Previous biomarker discovery studies in patients with chronic *T. cruzi* have mostly focused on amino acidic metabolites, found to be altered to different degrees in symptomatic and asymptomatic participants [10, 11]. While we observed similar changes in unadjusted models (Fig. 1a), only changes in lipid metabolites remained evident in our cohort after accounting for differences in the sex and age of participants.

Lipid metabolism is central to the development of *T. cruzi* and the pathogenesis of CD. Parasite lipids are known to induce inflammatory responses [24] and thrombosis [25]. It is also thought that accumulation of cholesterol, long-chain FAs, or phospholipids leads to increased oxidative stress in parasitized cells [26, 27].

A previous study has described changes in steroidogenesis in animals and humans infected with *T. cruzi* [10]. Changes in carnitine abundance and FA oxidation have been described in the hearts of patients with Chagas cardiomyopathy [12]. All these findings suggest that changes in the host’s lipidome might reflect pathological phenomena taking place in parasitized tissues.

Although limited by a small sample size (28 infected participants and 15 controls), the description of only relative abundance data, and pending further validation in an independent cohort, our results support the idea that altered lipid metabolism plays an important role in the pathogenesis of CD.

We detected statistically significant increases in two PEs in symptomatic participants: PE(18:0/20:4) and PE(18:1/20:4). Glycerophospholipids are the most abundant component of lipid membranes in eukaryotes, with PEs being the second most abundant type in *T. cruzi* amastigotes, representing 13% of the parasite’s total lipids [24]. The moiety of PEs depends on the nature of FAs associated with the hydrophilic head [28]. In *T. cruzi* PEs, these FAs can be either scavenged from the host cell or synthesized by the parasites. De novo FA synthesis in trypanosomatids relies on the action of enzymes of the elongase (ELO) family [24, 28]. The C18:1, C18:0, and C20:4 moieties observed in this study can be produced by ELO enzymes, and have been observed in PEs or lyso-PEs from *T. cruzi*. However, they are generally more abundant in epimastigotes than in tissue-dwelling forms [29], which along with the low parasitaemia observed in chronic CD suggests that the PEs identified are host-derived.

PEs are also a key component of mammalian cell membranes and participate in a wide variety of biological processes. Interestingly, increases have been described in the serum of patients with heart failure [30] as well as in mice with cardiac remodelling following ischemic disease [31]. Furthermore, PE(18:0/20:4) and PE(18:1/20:4) have been identified in the blood vessels of atherosclerotic mice [32] and in the plasma of humans with aortic dissection [33], suggesting a possible association with cardiovascular damage, as it has been proposed that increases in glycerophospholipids with long and unsaturated FAs could be a delayed adaptative response to tissue ischaemia [34].

Furthermore, increases in glycerophospholipids and their derivatives have been described in the hearts and digestive tracts of mice acutely infected with *T. cruzi* Y and CL Brener strains [26, 35]. Increases in lysoPE, a product of PE metabolism, were also detected in the hearts of *T. cruzi*-infected animals [26] and humans [12]. A recent metabolomic study [11] also found PEs with 18:0/20:4 and 18:1/20:4 moieties to be increased in patients with chronic *T. cruzi* infection, although more clearly in those in the indeterminate (asymptomatic) stage, which could be explained by differences in the processing and extraction of samples, the staging and treatment of the cohorts, the parasite strains involved in the infection, or some other host-derived confounders. It should be noted that none of these studies were designed to determine the exact *sn* position of the acyl groups of these molecules, which might be different from the one putatively assigned.

Asymptomatic participants in our cohort also had significant increases in the abundance of 10-hydroxydecanoic acid (also known as 10-hydroxycapric acid), an unsaturated FA with anti-inflammatory and immunomodulatory properties [36]. This finding contrasts with reductions previously described in patients with CD [10]. However, 10-hydroxydecanoic acid is a common component of cosmetics and dietary supplements [37], which suggests an exogenous origin and might also explain the observed discrepancies, limiting its potential utility as a biomarker despite the good discriminatory capacity observed in our study.

We also observed changes in the abundance of 28 metabolites following anti-parasitic treatment. Most of these seemed to be due to the direct effect of the medication on patient metabolism, as evidenced by the important differences between post-treatment samples and untreated controls (Additional file 6). Nonetheless sphingolipids such as sphinganine, hexadecasphinganine, and 4-HS were increased in the untreated asymptomatic group when compared with controls, but not after receiving treatment, when their abundance was significantly reduced (Fig. 4).

Reductions in sphingolipid abundance following treatment with benznidazole have been previously described in vitro in *T. cruzi*-infected myoblasts, although problems with the precise annotation of the molecules limited the interpretation of this finding [38].

Sphinganine (dihydrosphingosine, or DHS) is a long-chain base (LCB) involved in the synthesis of ceramides. Although produced by mammalian cells, it is less abundant than sphingosine, a different LCB [39]. Sphinganine plays a key role in the synthesis of inositol phosphorylceramide (IPC), a major anchor for various important surface proteins expressed in the different life stages of *T. cruzi*, including tissue-dwelling amastigotes and bloodstream trypomastigotes [40, 41].

In eukaryotes, sphinganine is also used to produce DHS-1-phosphate (DHS-1P) by sphingosine kinases [41]. DHS-1-P and the better studied sphingosine-1-phosphate (S1P) are well-known signalling molecules involved in downstream immune cell activation and cytokine production [42]. A signalling function for unmodified sphinganine has also been recently described, and linked with modulation of the unfolded protein response (UPR) [43], a mechanism of cell stress triggered by the endoplasmic reticulum and observed in several conditions, including animal models of Chagas cardiomyopathy [44].

Another sphingolipid identified, 4-HS (or phytosphingosine) is an LCB, which in yeast and some kinetoplastids is produced by hydroxylation of sphinganine at C-4 and can also be used as backbone for the synthesis of IPC [41, 45]. A recent study of the sphingolipidome of pathogenic kinetoplastids identified ceramides containing 4-HS in several *Trypanosoma* species, including *T. cruzi*, although these were particularly enriched in salivary species, such as *Trypanosoma brucei* [46]. In mammals, 4-HS has been identified only in specific tissues, such as the epidermis, the small intestine, and the kidneys [39]. Hexadecasphinganine, a shorter LCB that also decreased after treatment with benznidazole, is known to be depleted in *T. brucei* cultures exposed to high doses of nifurtimox [47].

In addition to these three molecules, we found similar changes in *N*-(tetradecanoyl)-sphinganine (a dihydroceramide) and 2S-(dimethylamino)-1,3R-octadecanediol (SP dimethyl, amino(18:0/2:0), also known as dimethylsphinganine). Interestingly, dimethylsphinganine increases during in vitro replication of *Leishmania donovani* promastigotes [48], and a structurally similar lipid, dimethylsphingosine, is a well-known sphingosine kinase inhibitor that reduces S1P and DHS-1P production, and has shown beneficial effects in animal models of *T. cruzi* cardiomyopathy, suggesting a possible role of this pathway in *T. cruzi* infection and treatment [49].

Sphingolipids are well-known mediators capable of modulating immune responses in a complex way [50], and their metabolism plays an important role in the progression of several infectious diseases [42, 51]. Given the low parasitaemia observed in chronic infection, most of these molecules are expected to come from the host. It also seems possible that changes observed in our cohort reflect a modulatory effect on tissue inflammation caused by anti-parasitic treatment, as has been suggested in previous studies [52, 53]. Nonetheless, the previously described role of some of the identified sphingolipids in parasite metabolism suggests that a link between their abundance and the presence of parasites in infected tissues cannot be entirely ruled out.

Validation of these findings in independent cohorts and a description of their mechanistic nature warrants further research. Additionally, despite important advances in the recent development of alternative chromatographic techniques for lipidomic analysis [54–56] and their incorporation into cost-effective microfluidic devices [57], regular access to the infrastructure needed to measure these metabolites in endemic countries would still limit their practical utility, even after successful validation. Nevertheless, despite these limitations, the fact that reductions in LCB abundance were observed in asymptomatic patients who cleared parasitaemia, and months after the completion of anti-parasitic treatment (Fig. 4), is interesting, as it suggests an as-yet undescribed role of sphingolipid metabolism in the pathogenesis of CD. Furthermore, such reduction highlights its appeal for the identification of biomarkers to monitor the response to antiparasitic treatment.

## Conclusions

Changes in PE and sphingolipid abundance show promise as potential tools for monitoring disease progression and treatment response in chronic *T. cruzi* infection, pending thorough validation of their clinical significance in independent cohorts.

## Supplementary Information

Additional file 1: Dataset 1: Covariate-adjusted results of the multiple linear regression analysis of the metabolomic study.

Additional file 2: Text S1: Comparisons between study groups based on treatment status. Figure S1: Differentially abundant metabolites across the study clinical groups before anti-parasitic treatment. Figure S2: Differentially abundant metabolites across the study clinical groups after anti-parasitic treatment.

Additional file 3: Dataset 2: Covariate-adjusted results of the multiple linear regression analysis of the metabolomic and lipidomic study disaggregated by treatment status.

Additional file 4: Dataset 3: Covariate-adjusted results of the multiple linear regression analysis of the lipidomic study.

Additional file 5: Text S2: Performance evaluation of the PLS-DA classification algorithm used in the multivariate ROC analysis. Figure S3: Performance validation of the PLS-DA classification model.

Additional file 6: Text S3: Analysis of the differences within the clinical groups following anti-parasitic treatment. Table S1: Metabolic features differentially detected in samples following anti-parasitic treatment (post-treatment vs pre-treatment) within the same clinical group. Figure S4: Box-and-whisker plots of differentially abundant metabolites based on treatment status in controls. Text S4: Differentially abundant features in samples with detectable parasitaemia via rtPCR. Table S2: Metabolic features differentially detected in samples with rtPCR+ versus rtPCR− *T. cruzi* infection. Figure S5: Metabolites differentially abundant based on the rtPCR status.

Additional file 7: Dataset 4: Data values used to plot figures presented in the manuscript.

## Data Availability

The complete results of the covariate-adjusted multiple linear regressions used to compare the different clinical groups described in this paper are presented as additional files. The original metabolomic and lipidomic datasets, along with the code used to process them and create the figures, is available at https://github.com/isglobal-chagas/Metabolomics-ChagasPath.git. Additionally, the data used to plot the figures presented in this manuscript are provided in Additional file 7.

## Abbreviations

4-HS: 4-Hydroxysphinganine
ANOVA: One-way analysis of variance
AUC: Area under the curve
CD: Chagas disease
CV: Cross validation
DHS: Dihydrosphingosine
DHS-1P: Dihydroxysphingosine 1-phosphate
EDTA: Ethylenediaminetetraacetic acid
ELISA: Enzyme-linked immunosorbent assay
FA: Fatty acid
FC: Fold change
FDR: False discovery rate
IPC: Inositol phosphorylceramide
LC/MS: Liquid chromatography/mass spectrometry
LCB: Long-chain base
LogFC: Logarithm base 2 fold change
PC: Phosphatidylcholine
PE: Phosphatidylethanolamine
PLS-DA: Partial least-squares discriminant analysis
ROC: Receiver operating characteristic
rtPCR: Real-time polymerase chain reaction
S1P: Sphingosine 1-phosphate
SD: Standard deviation
UHPLC: Ultra-high-performance liquid chromatography
UPR: Unfolded protein response
XCMS: eXtensible Computational Mass Spectrometry

## References

[1] Pérez-Molina JA, Molina I. Chagas disease. Lancet. 2018;391:82–94. 28673423 10.1016/S0140-6736(17)31612-4

[2] Ribeiro HG, Galdino OA, de Souza KSC, Rosa Neta AP, Lin-Wang HT, Cunha-Neto E, et al. Unraveling the role of miRNAs as biomarkers in Chagas cardiomyopathy: Insights into molecular pathophysiology. PLoS Negl Trop Dis. 2024;18:e0011865. 38300899 10.1371/journal.pntd.0011865 PMC10833550

[3] Sousa GR, Gomes JAS, Fares RCG, Damásio MPDS, Chaves AT, Ferreira KS, et al. Plasma cytokine expression is associated with cardiac morbidity in Chagas disease. PLoS ONE. 2014;9:e87082. 24603474 10.1371/journal.pone.0087082 PMC3945957

[4] Keating SM, Deng X, Fernandes F, Cunha-Neto E, Ribeiro AL, Adesina B, et al. Inflammatory and cardiac biomarkers are differentially expressed in clinical stages of Chagas disease. Int J Cardiol. 2015;199:451–9. 26277551 10.1016/j.ijcard.2015.07.040 PMC4868386

[5] Alonso-Padilla J, López MC, Esteva M, Zrein M, Casellas A, Gómez I, et al. Serological reactivity against *T. cruzi*-derived antigens: evaluation of their suitability for the assessment of response to treatment in chronic Chagas disease. Acta Trop. 2021;221:105990. 34090864 10.1016/j.actatropica.2021.105990

[6] Muñoz G, Vergara C, Martínez G, Apt W, Zulantay I. Quantification of immunoglobulin G against *Trypanosoma cruzi* in individuals with chronic Chagas disease treated with nifurtimox and evaluated in prolonged follow-up. Korean J Parasitol. 2019;57:39. 30840798 10.3347/kjp.2019.57.1.39 PMC6409214

[7] Fabbro DL, Streiger ML, Arias ED, Bizai ML, Del Barco M, Amicone NA. Trypanocide treatment among adults with chronic Chagas disease living in Santa Fe city (Argentina), over a mean follow-up of 21 years: parasitological, serological and clinical evolution. Rev Soc Bras Med Trop. 2007;40:1–10. 17486245 10.1590/s0037-86822007000100001

[8] Alonso-Padilla J, Abril M, de Noya BA, Almeida IC, Angheben A, Jorge TA, et al. Target product profile for a test for the early assessment of treatment efficacy in Chagas disease patients: an expert consensus. PLoS Negl Trop Dis. 2020;14:1–10. 10.1371/journal.pntd.0008035 PMC7179829 32324735

[9] Liu Z, Ulrich Vonbargen R, Mccall L-I, Burleigh B, Mcconville M. Central role of metabolism in *Trypanosoma cruzi* tropism and Chagas disease pathogenesis. Curr Opin Microbiol. 2021;63:204–9. 34455304 10.1016/j.mib.2021.07.015 PMC8463485

[10] Golizeh M, Nam J, Chatelain E, Jackson Y, Ohlund LB, Rasoolizadeh A, et al. New metabolic signature for Chagas disease reveals sex steroid perturbation in humans and mice. Heliyon. 2022;8:e12380. 36590505 10.1016/j.heliyon.2022.e12380 PMC9800200

[11] Herreros-Cabello A, Bosch-Nicolau P, Pérez-Molina JA, Salvador F, Monge-Maillo B, Rodriguez-Palomares JF, et al. Identification of Chagas disease biomarkers using untargeted metabolomics. Sci Rep. 2024;14:1–11. 39138245 10.1038/s41598-024-69205-w PMC11322173

[12] Díaz ML, Burgess K, Burchmore R, Gómez MA, Gómez-Ochoa SA, Echeverría LE, et al. Metabolomic profiling of end-stage heart failure secondary to chronic Chagas cardiomyopathy. Int J Mol Sci. 2022;23:10456. 36142367 10.3390/ijms231810456 PMC9499603

[13] Duffy T, Cura CI, Ramirez JC, Abate T, Cayo NM, Parrado R, et al. Analytical performance of a multiplex real-time PCR assay using TaqMan probes for quantification of *Trypanosoma cruzi* satellite DNA in blood samples. PLoS Negl Trop Dis. 2013;7:e2000. 23350002 10.1371/journal.pntd.0002000 PMC3547845

[14] Adusumilli R, Mallick P. Data conversion with ProteoWizard msConvert. Methods Mol Biol. 2017;1550:339–68. 28188540 10.1007/978-1-4939-6747-6_23

[15] Gloaguen Y, Morton F, Daly R, Gurden R, Rogers S, Wandy J, et al. PiMP my metabolome: an integrated, web-based tool for LC-MS metabolomics data. Bioinformatics. 2017;33:4007. 28961954 10.1093/bioinformatics/btx499 PMC5860087

[16] Smith CA, Want EJ, O’Maille G, Abagyan R, Siuzdak G. XCMS: Processing mass spectrometry data for metabolite profiling using nonlinear peak alignment, matching, and identification. Anal Chem. 2006;78:779–87. 16448051 10.1021/ac051437y

[17] Giné R, Capellades J, Badia JM, Vughs D, Schwaiger-Haber M, Alexandrov T, et al. HERMES: a molecular-formula-oriented method to target the metabolome. Nat Methods. 2021;18:1370–6. 34725482 10.1038/s41592-021-01307-z PMC9284938

[18] Pang Z, Chong J, Zhou G, De Lima Morais DA, Chang L, Barrette M, et al. MetaboAnalyst 5.0: narrowing the gap between raw spectra and functional insights. Nucl Acids Res. 2021;49:W388–96. 34019663 10.1093/nar/gkab382 PMC8265181

[19] Szymańska E, Saccenti E, Smilde AK, Westerhuis JA. Double-check: validation of diagnostic statistics for PLS-DA models in metabolomics studies. Metabolomics. 2012;8:3. 22593721 10.1007/s11306-011-0330-3 PMC3337399

[20] Ritchie ME, Phipson B, Wu D, Hu Y, Law CW, Shi W, et al. limma powers differential expression analyses for RNA-sequencing and microarray studies. Nucl Acids Res. 2015;43:e47–e47. 25605792 10.1093/nar/gkv007 PMC4402510

[21] Wickham H. ggplot2: Elegant Graphics for Data Analysis. Springer-Verlag New York. 2016.

[22] Bijlsma S, Bobeldijk I, Verheij ER, Ramaker R, Kochhar S, Macdonald IA, et al. Large-scale human metabolomics studies: a strategy for data (pre-) processing and validation. Anal Chem. 2006;78:567–74. 16408941 10.1021/ac051495j

[23] Ros-Lucas A, Gabaldón-Figueira JC, Martinez-Peinado N, Losada-Galvan I, Posada E, Escabia E, et al. Transcriptomic evidence of immune modulation in subjects with chronic *Trypanosoma cruzi* infection. J Infect Dis. 2024;jiae429. 10.1093/infdis/jiae429. 39194054 10.1093/infdis/jiae429

[24] Booth LA, Smith TK. Lipid metabolism in *Trypanosoma cruzi*: a review. Mol Biochem Parasitol. 2020;240:111324. 32961207 10.1016/j.molbiopara.2020.111324

[25] Gazos-Lopes F, Oliveira MM, Hoelz LVB, Vieira DP, Marques AF, Nakayasu ES, et al. Structural and functional analysis of a platelet-activating lysophosphatidylcholine of *Trypanosoma cruzi*. PLoS Negl Trop Dis. 2014;8:e3077. 25101628 10.1371/journal.pntd.0003077 PMC4125143

[26] Gironès N, Carbajosa S, Guerrero NA, Poveda C, Chillón-Marinas C, Fresno M. Global metabolomic profiling of acute myocarditis caused by *Trypanosoma cruzi* infection. PLoS Negl Trop Dis. 2014;8:e3337. 25412247 10.1371/journal.pntd.0003337 PMC4239010

[27] Johndrow C, Nelson R, Tanowitz H, Weiss LM, Nagajyothi F. *Trypanosoma cruzi* infection results in an increase in intracellular cholesterol. Microbes Infect. 2014;16:337. 24486184 10.1016/j.micinf.2014.01.001 PMC3999293

[28] de Aquino GP, Gomes MAM, Salinas RK, Laranjeira-Silva MF. Lipid and fatty acid metabolism in trypanosomatids. Microb Cell. 2021;8:262–75. 34782859 10.15698/mic2021.11.764 PMC8561143

[29] Harris M. Phospholipid analysis in *Trypanosoma cruzi*. Open Access Theses & Dissertations. 2011.

[30] Rong J, He T, Zhang J, Bai Z, Shi B. Serum lipidomics reveals phosphatidylethanolamine and phosphatidylcholine disorders in patients with myocardial infarction and post-myocardial infarction-heart failure. Lipids Health Dis. 2023;22:1–17. 37210547 10.1186/s12944-023-01832-0 PMC10199531

[31] Gowda SGB, Gowda D, Hou F, Chiba H, Parcha V, Arora P, et al. Temporal lipid profiling in the progression from acute to chronic heart failure in mice and ischemic human hearts. Atherosclerosis. 2022;363:30–41. 36455306 10.1016/j.atherosclerosis.2022.11.005

[32] Zhang L, Xiong L, Fan L, Diao H, Tang M, Luo E, et al. Vascular lipidomics analysis reveales increased levels of phosphocholine and lysophosphocholine in atherosclerotic mice. Nutr Metab. 2023;20:1. 10.1186/s12986-022-00723-y PMC9811766 36600244

[33] Huang H, Ye G, Lai SQ, Zou HX, Yuan B, Wu QC, et al. Plasma lipidomics identifies unique lipid signatures and potential biomarkers for patients with aortic dissection. Front Cardiovasc Med. 2021;8:757022. 34778409 10.3389/fcvm.2021.757022 PMC8581228

[34] Afshinnia F, Jadoon A, Rajendiran TM, Soni T, Byun J, Michailidis G, et al. Plasma lipidomic profiling identifies a novel complex lipid signature associated with ischemic stroke in chronic kidney disease. J Transl Sci. 2020;6:419. 33240530 PMC7682927

[35] Dean DA, Gautham G, Siqueira-Neto JL, McKerrow JH, Dorrestein PC, McCall LI. Spatial metabolomics identifies localized chemical changes in heart tissue during chronic cardiac Chagas disease. PLoS Negl Trop Dis. 2021;15:e0009819. 34606502 10.1371/journal.pntd.0009819 PMC8516257

[36] You M, Miao Z, Sienkiewicz O, Jiang X, Zhao X, Hu F. 10-Hydroxydecanoic acid inhibits LPS-induced inflammation by targeting p53 in microglial cells. Int Immunopharmacol. 2020;84:106501. 32311670 10.1016/j.intimp.2020.106501

[37] Atef B, Ishak RAH, Badawy SS, Osman R. 10-Hydroxy decanoic acid-based vesicles as a novel topical delivery system: would it be a better platform than conventional oleic acid ufasomes for skin cancer treatment? Pharmaceutics. 2023;15:1461. 37242703 10.3390/pharmaceutics15051461 PMC10223426

[38] Hennig K, Abi-Ghanem J, Bunescu A, Meniche X, Biliaut E, Ouattara AD, et al. Metabolomics, lipidomics and proteomics profiling of myoblasts infected with *Trypanosoma cruzi* after treatment with different drugs against Chagas disease. Metabolomics. 2019;15:1–12. 10.1007/s11306-019-1583-5 31440849

[39] Kitamura T, Seki N, Kihara A. Phytosphingosine degradation pathway includes fatty acid α-oxidation reactions in the endoplasmic reticulum. Proc Natl Acad Sci USA. 2017;114:E2616–23. 28289220 10.1073/pnas.1700138114 PMC5380038

[40] De Lederkremer RM, Agusti R, Docampo R. Inositolphosphoceramide metabolism in *Trypanosoma cruzi* as compared with other trypanosomatids. J Eukaryot Microbiol. 2011;58:79–87. 21332877 10.1111/j.1550-7408.2011.00533.x PMC3444516

[41] Koeller CME, Heise N. The sphingolipid biosynthetic pathway is a potential target for chemotherapy against Chagas disease. Enzyme Res. 2011;648159:13. 10.4061/2011/648159. 21603271 10.4061/2011/648159 PMC3092604

[42] Schuurman AR, Chouchane O, Butler JM, Peters-Sengers H, Joosten S, Brands X, et al. The shifting lipidomic landscape of blood monocytes and neutrophils during pneumonia. JCI Insight. 2024;9:e164400. 38385743 10.1172/jci.insight.164400 PMC10967382

[43] Farley S, Stein F, Haberkant P, Tafesse FG, Schultz C. Trifunctional sphinganine: a new tool to dissect sphingolipid function. ACS Chem Biol. 2024;19:336–47. 38284972 10.1021/acschembio.3c00554 PMC10878393

[44] Ayyappan JP, Lizardo K, Wang S, Yurkow E, Nagajyothi JF. Inhibition of ER stress by 2-aminopurine treatment modulates cardiomyopathy in a murine chronic Chagas disease model. Biomol Ther. 2019;27:386. 10.4062/biomolther.2018.193 PMC6609105 30879276

[45] Figueiredo JM, Rodrigues DC, Silva RCMC, Koeller CM, Jiang JC, Jazwinski SM, et al. Molecular and functional characterization of the ceramide synthase from *Trypanosoma cruzi*. Mol Biochem Parasitol. 2012;182:62. 22226824 10.1016/j.molbiopara.2011.12.006 PMC3551351

[46] Guan XL, Mäser P. Comparative sphingolipidomics of disease-causing trypanosomatids reveal unique lifecycle- and taxonomy-specific lipid chemistries. Sci Rep. 2017;7:1–13. 29051559 10.1038/s41598-017-13931-x PMC5648825

[47] May Vincent I. Using metabolomic analyses to study mode of action of and resistance to eflornithine in *Trypanosoma brucei*. University of Glasgow; 2011.

[48] Silva AM, Cordeiro-da-Silva A, Coombs GH. Metabolic variation during development in culture of *Leishmania donovani* promastigotes. PLoS Negl Trop Dis. 2011;5:e1451. 22206037 10.1371/journal.pntd.0001451 PMC3243725

[49] Vasconcelos JF, Meira CS, Silva DN, Nonaka CKV, Daltro PS, MacAmbira SG, et al. Therapeutic effects of sphingosine kinase inhibitor N,N-dimethylsphingosine (DMS) in experimental chronic Chagas disease cardiomyopathy. Sci Rep. 2017;7:6171. 28733584 10.1038/s41598-017-06275-z PMC5522404

[50] Lee M, Lee SY, Bae YS. Functional roles of sphingolipids in immunity and their implication in disease. Exp Mol Med. 2023;55:1110. 37258585 10.1038/s12276-023-01018-9 PMC10318102

[51] Wang J, Chen YL, Li YK, Chen DK, He JF, Yao N. Functions of sphingolipids in pathogenesis during host-pathogen interactions. Front Microbiol. 2021;12:701041. 34408731 10.3389/fmicb.2021.701041 PMC8366399

[52] Marañón C, Egui A, Fernández-Villegas A, Carrilero B, Thomas MC, Segovia M, et al. Benznidazole treatment reduces the induction of indoleamine 2,3-dioxygenase (IDO) enzymatic activity in Chagas disease symptomatic patients. Parasite Immunol. 2013;35:180–7. 23473453 10.1111/pim.12030

[53] Pérez-Antón E, Egui A, Thomas MC, Puerta CJ, González JM, Cuéllar A, et al. Impact of benznidazole treatment on the functional response of *Trypanosoma cruzi* antigen-specific CD4+CD8+ T cells in chronic Chagas disease patients. PLoS Negl Trop Dis. 2018;12:e0006480. 29750791 10.1371/journal.pntd.0006480 PMC5965897

[54] Sancho-Albero M, Jarne C, Savirón M, Martín-Duque P, Membrado L, Cebolla VL, et al. High-performance thin-layer chromatography-densitometry-tandem ESI-MS to evaluate phospholipid content in exosomes of cancer cells. Int J Mol Sci. 2022;23:1150. 35163074 10.3390/ijms23031150 PMC8835402

[55] Jarne C, Membrado L, Savirón M, Vela J, Orduna J, Garriga R, et al. Globotriaosylceramide-related biomarkers of fabry disease identified in plasma by high-performance thin-layer chromatography-densitometry-mass spectrometry. J Chromatogr A. 2021;1638:461895. 33477028 10.1016/j.chroma.2021.461895

[56] Uranbileg B, Sakai E, Kubota M, Isago H, Sumitani M, Yatomi Y, et al. Development of an advanced liquid chromatography–tandem mass spectrometry measurement system for simultaneous sphingolipid analysis. Sci Rep. 2024;14:1–13. 38459112 10.1038/s41598-024-56321-w PMC10923881

[57] Davic A, Cascio M. Development of a microfluidic platform for trace lipid analysis. Metabolites. 2021;11:1–19. 10.3390/metabo11030130 PMC7996208 33668377

