# Supplementary material for: Changes in lipid abundance are associated with disease progression and treatment response in chronic Trypanosoma cruzi infection
Source: Parasit Vectors. 2024 Nov 9;17:459. doi: 10.1186/s13071-024-06548-3 (PMC11549750; doi:10.1186/s13071-024-06548-3)
Supplement: Supplementary file 2 — Additional file 2: Text S1: Comparisons between study groups based on treatment status. Figure S1: Differentially abundant metabolites across the study clinical groups before anti-parasitic treatment. Figure S2: Differentially abundant metabolites across the study clinical groups after anti-parasitic treatment. [file 13071_2024_6548_MOESM2_ESM.pdf]

Additional file 2

Multiple linear regression comparing study groups based on their treatment status

In addition to the pooled data used to compare the abundance of different metabolites and lipids in the main analysis, we performed comparisons using the same multiple linear model on datasets disaggregated by the treatment status of participants. This model was also adjusted for age and sex.

Differences in untreated groups

In this analysis, we observed increases in 10-hydroxydecanoic acid, sphinganine and 4-hydroxysphinganine in untreated asymptomatic participants compared with controls (**Figure S1a**) and decreases in D-glucarate, in untreated symptomatic participants compared with asymptomatics (**Figure S1c**). Hexadecasphinganine also showed statistically significant increases in asymptomatic participants compared with controls ($p<0.001$), but the logFC was below the pre-defined threshold (logFC = 0.12). No significant differences were observed when comparing untreated symptomatic participants and controls (**Figure S1b**).

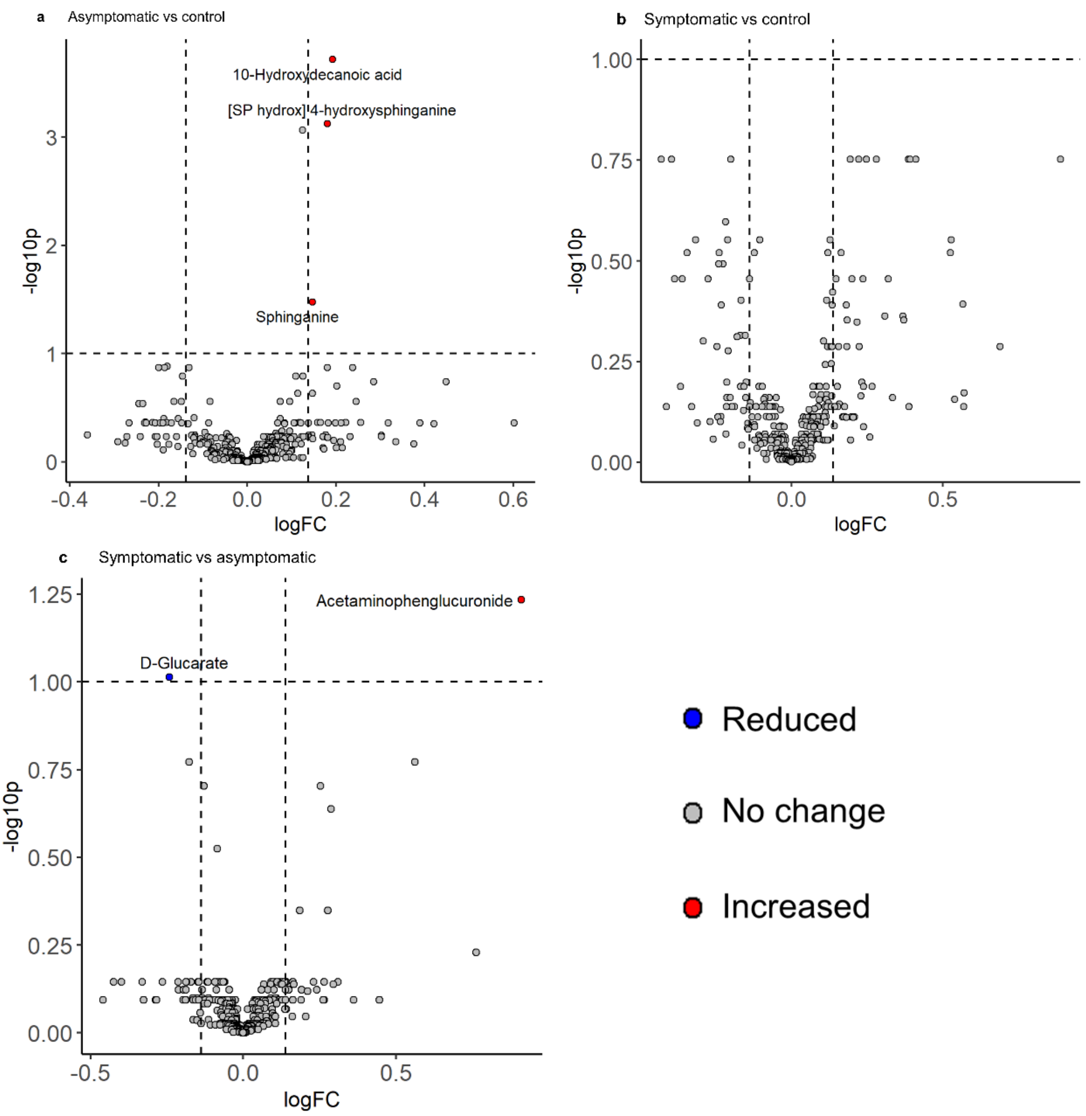

Figure S1. Differentially abundant metabolites across the study clinical groups before anti-parasitic treatment: The comparison of asymptomatic *vs* control subjects is shown in (a); that of symptomatic *vs* control in (b); and the symptomatic *vs* asymptomatic in (c). Metabolites were considered to be differentially expressed if the logFC was > ±0.138, and presented an FDR < 0.1. All *p*-values have been adjusted using the Benjamini Hochberg's method to control the FDR.

Differences in treated groups

Several metabolites showed differential abundance when comparing treated asymptomatic and symptomatic groups with controls (**Figure S2a-b**). However, this can probably be explained by the fact that controls did not receive any anti-parasitic

medication. Thus, these changes are likely caused by metabolic disturbances produced directly by anti-parasitic treatment.

Increases in PE 18:0/20:4, as well as 7-amynoethil-7-carbaguanine, and tetrahydrogeranyl-bacteriophytin were observed in treated symptomatics when compared with the asymptomatic group (**Figure S2c**). Similarly, 10-hydroxydecanoic acid was decreased in treated symptomatic participants.

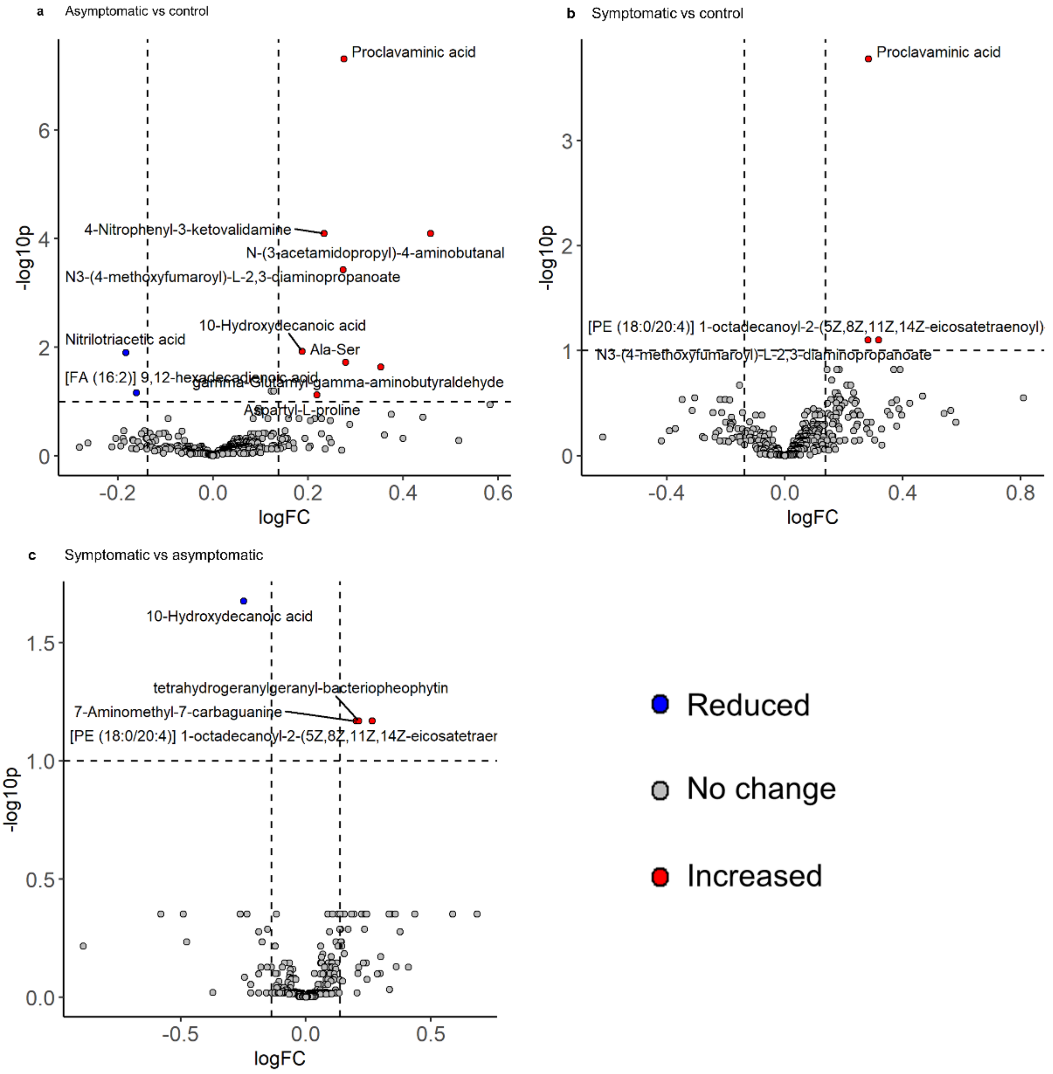

Figure S2. Differentially abundant metabolites in the different clinical groups after receiving anti-parasitic treatment. The comparison of asymptomatic *vs* control subjects

is shown in (a); that of symptomatic *vs* controls in (b); and the one between symptomatic and asymptomatic in (c). Metabolites were considered to be differentially expressed if the FC was > +/-1, and presented an FDR < 0.1. All *p*-values have been adjusted using the Benjamini Hochberg's method to control the FDR.

In the lipidomic analysis, the only inter-group difference observed was an increase in the positively charged form of PE (18:1/20:4), which was increased in untreated symptomatic participants compared with controls; further suggesting that changes described for this compound were not attributed to an effect of anti-parasitic treatment.

An excel spreadsheet with the full results of the multiple linear model with comparisons disaggregated by treatment status is provided in Supplementary file 3.
