# Supplementary material for: Changes in lipid abundance are associated with disease progression and treatment response in chronic Trypanosoma cruzi infection
Source: Parasit Vectors. 2024 Nov 9;17:459. doi: 10.1186/s13071-024-06548-3 (PMC11549750; doi:10.1186/s13071-024-06548-3)
Supplement: Supplementary file 5 — Additional file 5: Text S2: Performance evaluation of the PLS-DA classification algorithm used in the multivariate ROC analysis. Figure S3: Performance validation of the PLS-DA classification model. [file 13071_2024_6548_MOESM5_ESM.pdf]

Additional file 5

Performance evaluation of the PLS-DA classification algorithm used in the multivariate ROC analysis

A partial least squares discriminant analysis (PLS-DA) classification model to discriminate between symptomatic and asymptomatic participants was constructed using the Metaboanalyst 5.0 platform and using a two latent variable input. The model included all features that showed statistically significant differences in abundance between symptomatic and either asymptomatic or control groups:

- 10-hydroxydecanoic acid: identified in the general metabolomic analysis.
- Deprotonated and protonated forms of PE(18:1/20:4): identified in the lipidomic analysis.
- Deprotonated and protonated forms of PE(18:0/20:4): identified in the lipidomic analysis, and in the metabolomic analysis.

The predictive accuracy of the model was estimated using the cross-validation method (CV) using 100 permutations. PLS-DA models tend to overfit the data, leading to a scenario in which the model can discriminate between the two groups by random chance. To determine if this is the case, the group label (symptomatic or asymptomatic) is reassigned randomly (shuffled). The performance of the model is then evaluated using balanced sub-sampling cross validation, building the classifier model with 2/3 of the total data, and then evaluating its performance on the remaining 1/3.

The performance with shuffled data is then evaluated and recorded. This process was repeated 1,000 times, and the performance of the model using the original labels was compared with that obtained using the permuted labels. After sufficient permutations, the performance measures based on the shuffled data will form a normal distribution. If the performance score obtained when using the original data falls outside that distribution,

the result can be considered significant, and the model is assumed to differentiate between the two groups.

Performance validation of the model using the features described above produced a classifier with an area under the curve (AUC) of 0.88 (95% CI: 0.72-0.99), and a mean predictive accuracy of 0.8 (p = 0.003; **Figure S3**).

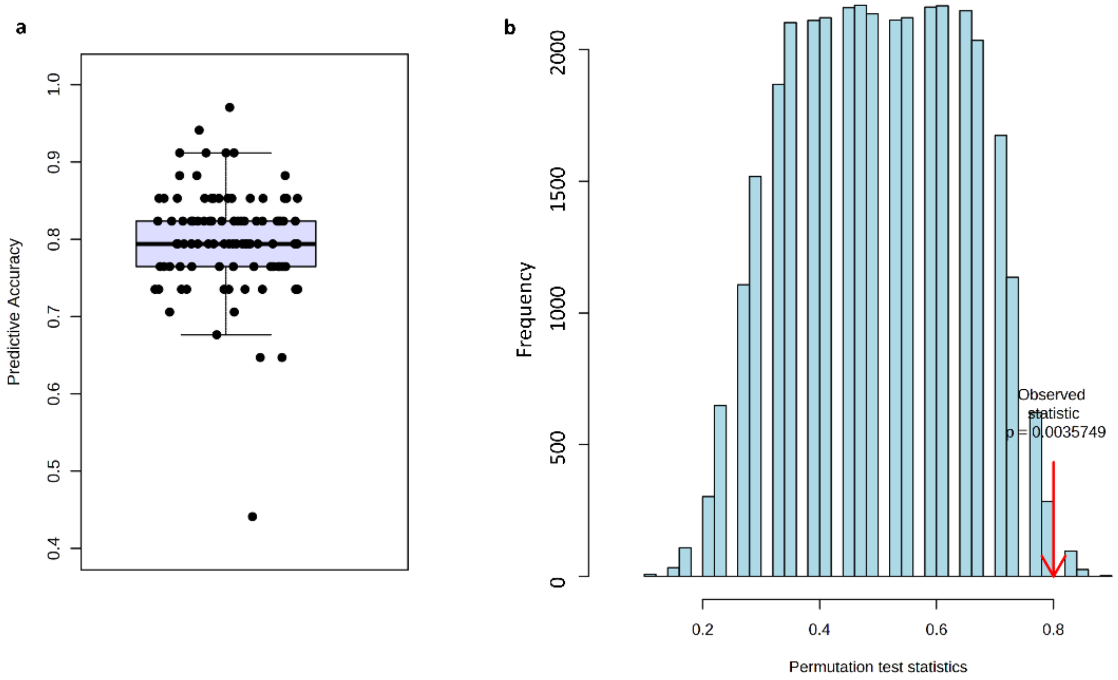

Figure S3. Performance evaluation of the multivariate PLS-DA classification model. Mean predictive accuracy of the model (b). Frequency distribution of predictive accuracy of the model upon 1,000 permutations (c).
