# Supplementary material for: Changes in lipid abundance are associated with disease progression and treatment response in chronic Trypanosoma cruzi infection
Source: Parasit Vectors. 2024 Nov 9;17:459. doi: 10.1186/s13071-024-06548-3 (PMC11549750; doi:10.1186/s13071-024-06548-3)
Supplement: Supplementary file 6 — Additional file 6: Text S3: Analysis of the differences within the clinical groups following anti-parasitic treatment. Table S1: Metabolic features differentially detected in samples following anti-parasitic treatment (post-treatment vs pre-treatment) within the same clinical group. Figure S4: Box-and-whisker plots of differentially abundant metabolites based on treatment status in controls. Text S4: Differentially abundant features in samples with detectable parasitaemia via rtPCR. Table S2: Metabolic features differentially detected in samples with rtPCR+ versus rtPCR− T. cruzi infection. Figure S5: Metabolites differentially abundant based on the rtPCR status. [file 13071_2024_6548_MOESM6_ESM.pdf]

Additional file 6

Analysis of the differences within the clinical groups following anti-parasitic treatment

The metabolomic profile of participants before and after receiving anti-parasitic therapy was evaluated using a paired t-test adjusted for multiple testing. Since each pair of samples came from the same participants no further covariate adjustment was performed. None of the features shown to discriminate between symptomatics and the rest of the groups showed differences associated with treatment.

In asymptomatic participants, we observed significant increases (logFC $\geq +0.138$, $p < 0.1$) in 20 metabolites including proclavaminic acid, gamma-Glutamyl-gamma-aminobutyraldehyde, N-(3-acetamidopropyl)-4-aminobutanal, N-hydroxy-N-acetylcadaverine, as well as oligopeptides like Lys-Val-Val, Ala-Ser, Aspartyl-L-Proline, and Glu-Thr. Two negatively charged lipids, PI 38:4 and PI 40:5, were also elevated following treatment. Proclavaminic acid was the only feature also significantly increased in symptomatic participants following treatment. Significant decreases (FC ≤ -0.138, $p < 0.1$) were observed in eight metabolic features: Nitriloacetic acid, N-eicosanoyl-ethanolamine (FA 20:0), sucrose, and five sphingolipids: 4-hydroxysphinganine, hexadecasphinganine, sphinganine, SP dimethyl,amino(18:0/2:0) and [SP (14:0)] N-(tetradecanoyl)-sphinganine (**Table S1**, **Figure S4**). The only sphingolipid that increased after treatment was SM(d18:0/18:1).

Table S1. Metabolic features differentially detected in samples following anti-parasitic treatment (post-treatment vs pre-treatment) within the same clinical grup.

MM (g/mol)	RT (seconds)	Putative annotation	Formula	logFC	FDR
**Untargeted metabolomic analysis**					
**Asymptomatics**					
202.10	591.72	Proclavaminic acid	$C_8H_{14}N_2O_4$	↑ 0.87	<0.001
191.04	670.93	Nitrilotriacetic acid	$C_6H_9NO_6$	↓ -0.48	<0.001
317.29	249.78	4-hydroxysphinganine	$C_{18}H_{39}NO_3$	↓ -0.40	<0.001
273.27	268.39	Hexadecasphinganine	$C_{16}H_{35}NO_2$	↓ -0.33	0.001
355.34	226.98	FA (20:0) N-eicosanoyl-ethanolamine	$C_{22}H_{45}NO_2$	↓ -0.29	0.001
216.11	555.93	gamma-Glutamyl-gamma-aminobutyraldehyde	$C_9H_{16}N_2O_4$	↑ 2.02	0.001
186.14	439.32	N-(3-acetamidopropyl)-4-aminobutanal	$C_9H_{18}N_2O_2$	↑ 2.05	0.001
301.30	258.21	Sphinganine	$C_{18}H_{39}NO_2$	↓ -0.33	0.001
160.12	481.70	N-hydroxy-N-acetylcadaverine	$C_7H_{16}N_2O_2$	↑ 2.21	0.002
172.12	467.47	Lys-Val-Val	$C_{16}H_{32}N_4O_4$	↑ 1.44	0.002
296.10	633.31	4-Nitrophenyl-3-ketovalidamine	$C_{13}H_{16}N_2O_6$	↑ 1.26	0.002
446.38	175.55	13-hydroxy-alpha-tocopherol	$C_{29}H_{50}O_3$	↑ 0.84	0.003
176.08	651.80	Ala-Ser	$C_6H_{12}N_2O_4$	↑ 1.06	0.004
230.09	593.11	Aspartyl-L-proline	$C_9H_{14}N_2O_5$	↑ 0.78	0.004
368.34	276.05	9,10-seco-5,7,10(19)-cholestatriene	$C_{27}H_{44}$	↑ 0.25	0.009
428.36	174.85	Fucosterol epoxide	$C_{29}H_{48}O_2$	↑ 0.66	0.019
248.10	515.55	Glu-Thr	$C_9H_{16}N_2O_6$	↑ 0.27	0.019
216.07	623.12	N3-(4-methoxyfumaroyl)-L-2,3-diaminopropanoate	$C_8H_{12}N_2O_5$	↑ 0.74	0.021
329.33	240.48	SP dimethyl,amino(18:0/2:0)	$C_{20}H_{43}NO_2$	↓ -0.23	0.021
305.14	204.73	Endomorphin-1	$C_{34}H_{38}N_6O_5$	↑ 1.17	0.031
730.60	193.70	SM(d18:0/18:1)	$C_{41}H_{83}N_2O_6P$	↑ 0.31	0.063
342.12	685.07	Sucrose	$C_{12}H_{22}O_{11}$	↓ -1.24	0.064
511.50	179.30	[SP (14:0)] N-(tetradecanoyl)-sphinganine	$C_{32}H_{65}NO_3$	↓ -0.39	0.066
259.16	183.25	2-Heptyl-4-hydroxyquinoline-N-oxide	$C_{16}H_{21}NO_2$	↑ 0.41	0.080
835.61	179.95	PC (18:0/22:5)	$C_{48}H_{86}NO_8P$	↑ 0.24	0.087
71.07	586.26	3-Buten-1-amine	C_4H_9N	↑ 0.16	0.090
**Symptomatics**					
202.10	591.72	Proclavaminic acid	$C_8H_{14}N_2O_4$	↑ 0.89	0.092
**Targeted lipidomic analysis**					
**Negatively-charged lipids**					
**Asymptomatics**					
885.55	411.38	PI 38:4	$[C_{47}H_{82}O_{13}P]^-$	↑ 0.29	0.017
911.57	411.50	PI 40:5	$[C_{49}H_{84}O_{13}P]^-$	↑ 0.40	0.034

FA: Fatty acid. MM: Monoisotopic mass. RT: Retention time. SM: sphingomyelin, SP: sphingolipid. LogFC represents the log2 fold change in the mean abundance of a feature in the post- vs the pre-treatment group. *P*-values have been obtained using a paired t-test, and adjusted to account for multiple testing using the Benjamini Hochberg's method to control the FDR.

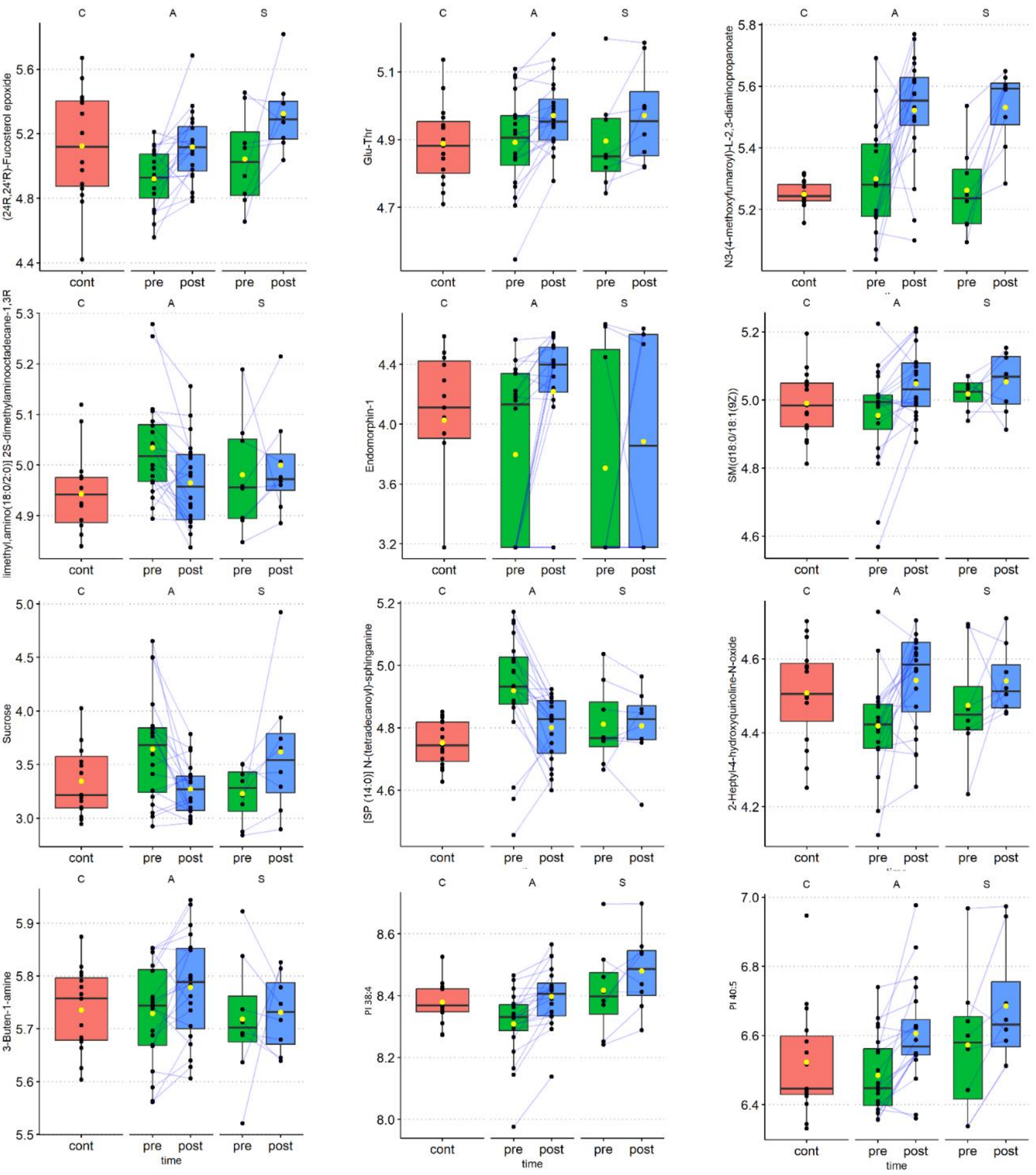

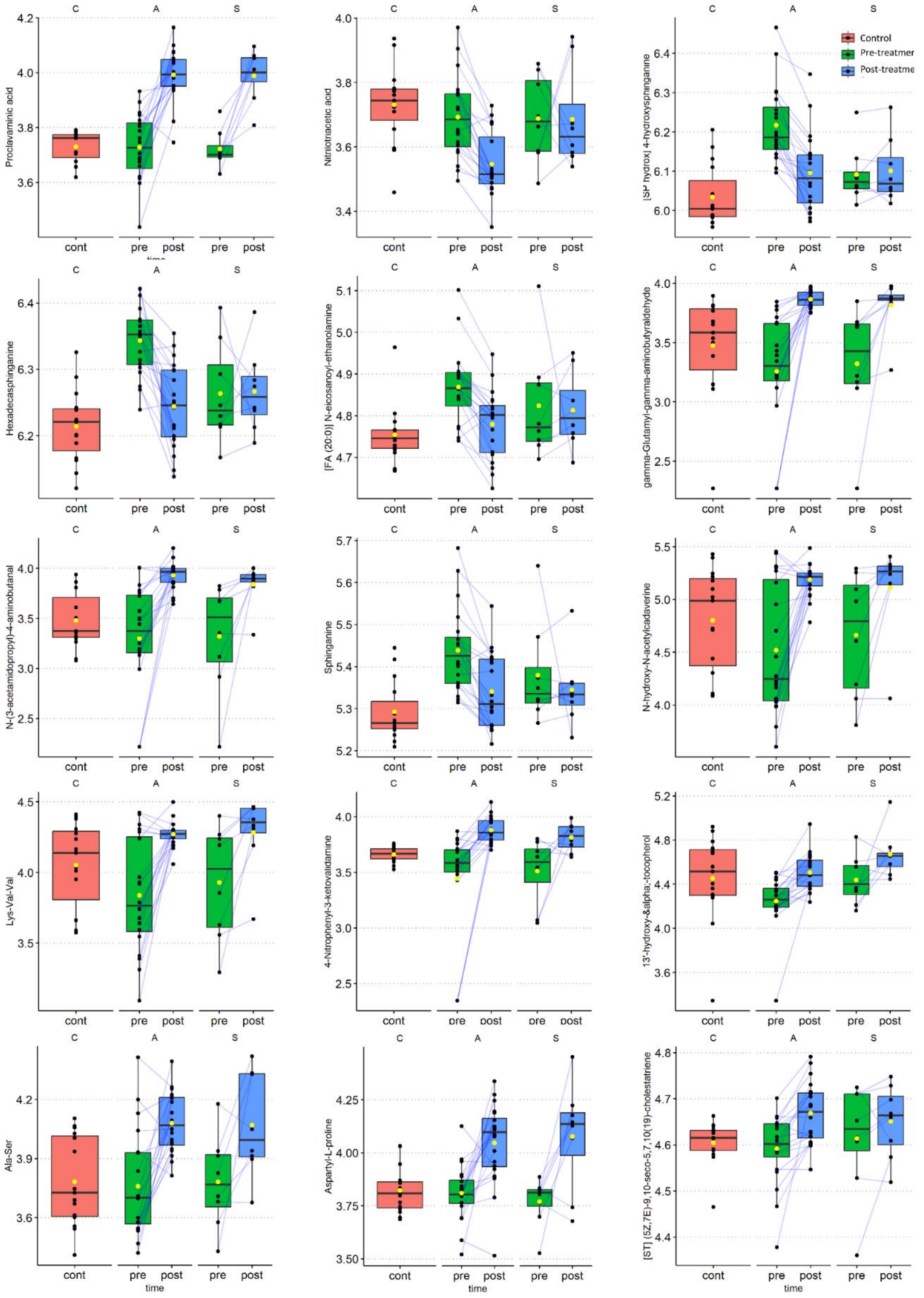

Figure S4. Box whisker plots of differentially abundant metabolites based on treatment status in in controls (C, 15 samples), asymptomatic (A, 20 samples pre- and post-treatment) and symptomatic groups (S, 8 samples pre- and post-treatment). Yellow dots represent group means.

Differentially abundant features in samples with detectable parasitaemia via rtPCR

Differences in the abundance of 12 metabolites were found in rtPCR+ samples, compared to negative ones (**Table S2, Figure S5**), with significant increases in [SP (14:0)] N-(tetradecanoyl)-sphinganine, N3-(4-methoxyfumaroyl)-L-2,3-diaminopropanoate, L-Aspartate, Glu-Gly, Aspartyl-L-proline, Endomorphin-1, 13'-hydroxy-α-tocopherol, N-gamma-Acetyldiaminobutyrate, [C10H21O4S2As2]-, (24R,24'R)-Fucosterol epoxide, 2-O-glutaroyl-1-O-palmitoyl-sn-glycero-3-phosphocholine(1-)], and PC(36:5). However, these differences were no longer significant after including the treatment status of the samples in the model. This was expected, considering the strong correlation between receiving anti-parasitic treatment and clearing parasitaemia (nine positive rtPCR samples were obtained before treatment, and only one after it, X^2= 6.8, df =1, p = 0.009).

Table S2. Metabolic features differentially detected in samples with rtPCR+ versus rtPCR- *T. cruzi* infection.

MM (g/mol)	RT (seconds)	Putative annotation	Formula	logFC	adj.P.Val
216.07	623.12	N3-(4-methoxyfumaroyl)-L-2,3-diaminopropanoate	$C_8H_{12}N_2O_5$	↓-0.26	0.023
133.04	645.97	L-Aspartate	$C_4H_7NO_4$	↓-0.23	0.031
204.07	586.92	Glu-Gly	$C_7H_{12}N_2O_5$	↓-0.56	0.034
305.14	204.73	Endomorphin-1	$C_{34}H_{38}N_6O_5$	↓-0.67	0.040
230.09	593.11	Aspartyl-L-proline	$C_9H_{14}N_2O_5$	↓-0.25	0.040
446.38	175.55	13'-hydroxy-α-tocopherol	$C_{29}H_{50}O_3$	↓-0.33	0.040
160.08	505.08	N-gamma-Acetyldiaminobutyrate	$C_6H_{12}N_2O_3$	↓-0.27	0.047
511.50	179.30	[SP (14:0)] N-(tetradecanoyl)-sphinganine	$C_{32}H_{65}NO_3$	↑0.15	0.067
428.36	174.85	(24R,24'R)-Fucosterol epoxide	$C_{29}H_{48}O_2$	↓-0.28	0.086
**Negatively charged**					
418.93	49.02	Not annotated	$[C_{10}H_{21}O_4S_2As_2]$-	↓-0.22	0.001
608.36	143.60	2-O-glutaroyl-1-O-palmitoyl-sn-glycero-3-phosphocholine	$[C_{29}H_{55}NO_{10}P]$-	↓-0.18	0.008
**Positively charged**					
780.55	408.74	PC(36:5)	$[C_{44}H_{79}NO_8P]$+	↓-0.28	0.016

MM: Monoisotopic mass. RT: Retention time. LogFC: represents the log2 fold change in the mean abundance of a feature in the rtPCR positive compared with the rtPCR- groups. Analysis controls for age, sex and the presence of comorbidities. *p*-values have been obtained using a multiple linear regression adjusted for age and sex, and treating participant ID as a random effect, to account for the inclusion of pre- and post-treatment samples; and account for multiple testing using the Benjamini Hochberg's method to control the FDR.

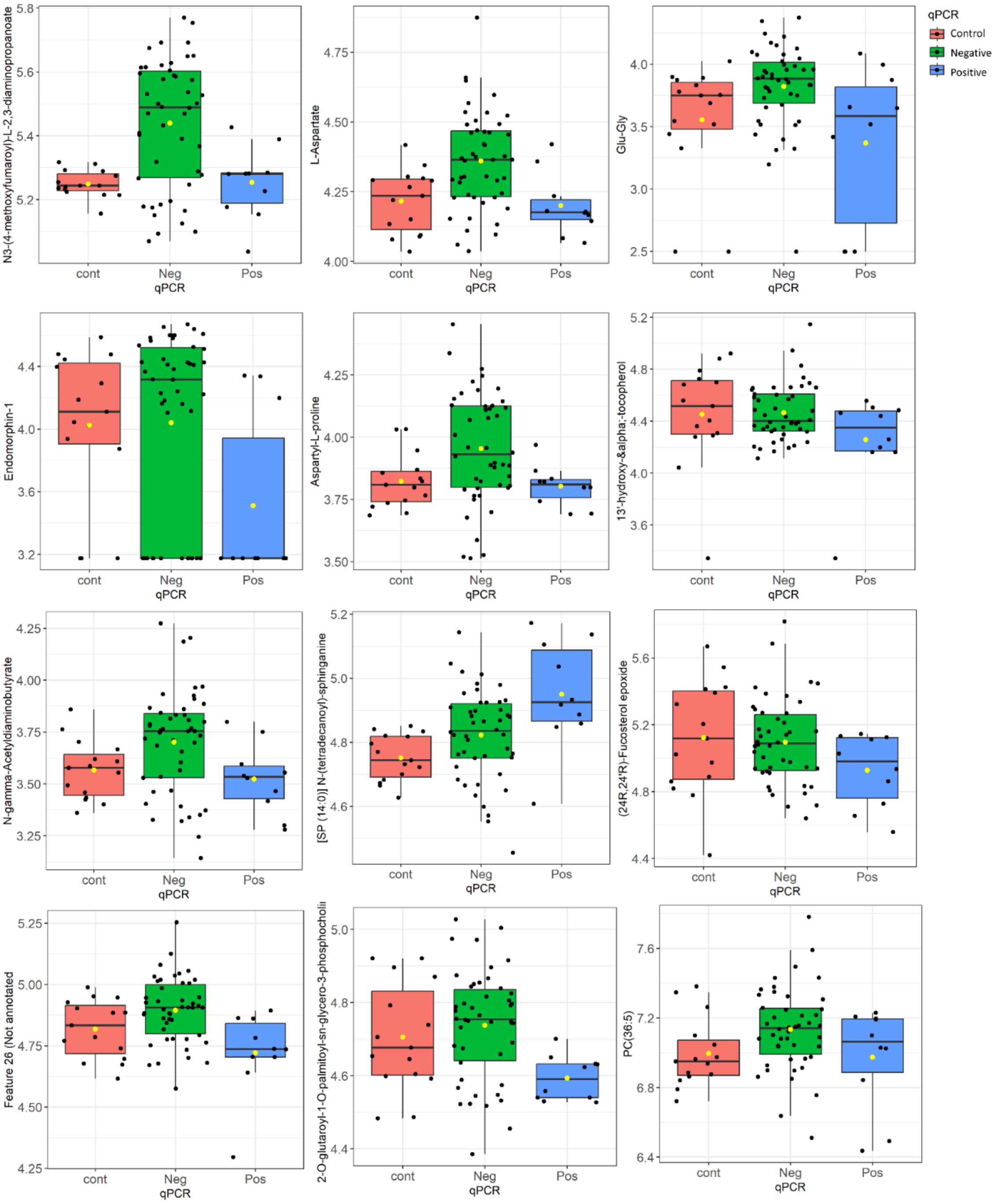

Figure S5. **Metabolites differentially abundant based on the rtPCR status.** Controls (n = 15 samples), negative (Neg) (n = 43 samples), positive (Pos) (n = 10 samples). Yellow dots represent group means.
